# Computational and molecular docking analysis of a novel azo compound and its nanocomposite biopolymer synthesized via a single-pot method

**DOI:** 10.1038/s41598-025-96866-y

**Published:** 2025-11-05

**Authors:** Manar Ghyath Abd-Almutalib, N. A. Naser

**Affiliations:** 1 Department of Pharmacy Techniques, Kufa, Technical Institute, Al-Furat Al-Awsat Technical University, 31001 Kufa, Al-Najaf, Iraq; 2 College of Pharmacy, Al-Mustaqbal University, 51001 Babylon, Hilla, Iraq; 3 Department of Chemistry, Faculty of Science, University of Kufa, Najaf Governorate, 21, Iraq

**Keywords:** Density Functional Theory (DFT), Azo compound, Molecular docking, Non-covalent interactions, Nanocomposite, Na-bentonite, Polycaprolactone (PCL), Glycerol, X-ray Diffraction (XRD), Transmission Electron Microscopy (TEM), Chemical modification, Computational chemistry

## Abstract

The synthesis of an azo molecule from phenol and dapsone was investigated using density functional theory (DFT) and infrared spectroscopy. The electronic structure and molecular interactions of the 4-((4-((4-aminophenyl)sulfonyl)phenyl)diazenyl) phenol (AZO) compound were analyzed at the B3LYP/6-311G(d,p) level. Pharmacokinetic properties were predicted using Swiss ADME, and molecular docking revealed π-alkyl and hydrophobic interactions with the *3H7O* protein. Structural analysis via XRD and TEM confirmed the successful intercalation of AZO into Na-BNT, contributing to improved dispersion within the PCL/cornstarch matrix. The resulting nanocomposite demonstrated enhanced thermal stability and mechanical performance, highlighting its potential for biodegradable packaging and advanced material applications.

## Introduction

The integration of high-performance computational tools has revolutionized pharmaceutical discovery by enabling precise molecular structure and electronic property analysis. Quantum simulations based on density functional theory (DFT) have provided deeper insights into molecular dynamics, thereby facilitating the design of safer and more effective therapeutic compounds. These computational strategies significantly reduce the cost and duration of empirical drug testing, accelerating the development of treatments for diseases such as cancer, COVID-19, and neurodegenerative disorders^1^ Gusarov, 2021

Azo compounds, characterized by the –N = N– functional group, have attracted considerable attention due to their pharmacological potential. These molecules exhibit strong binding affinities to protein receptors, making them promising candidates for drug development^2,3^. Although non-covalent interactions are weaker than covalent bonds, they play a crucial role in drug design, influencing molecular docking efficiency and enzymatic regulation^4^. Recent studies indicate that azo-based compounds demonstrate efficacy in treating parasitic infections, including scabies mites^5,6^. However, understanding their molecular behavior requires an integrated approach that combines computational and spectroscopic investigations^7,8^.

Beyond biomedical applications, azo compounds have been widely explored in materials science, particularly in the development of biodegradable polymers. The increasing demand for sustainable materials has driven research into polymer nanocomposites derived from renewable resources such as polycaprolactone (PCL) and starch-based composites^9–11^. However, challenges such as high stiffness and low molecular weight limit their practical applications. Various strategies, including plasticizer incorporation and peroxide modification, have been employed to enhance the mechanical, thermal, and degradation properties of PCL-based composites^12,13^. In particular, nanocomposite technology has proven to be an effective means of reinforcing biodegradable polymers. Studies have shown that even minimal organoclay filler (0.5–5%) addition can significantly enhance the structural integrity of these materials^14,15^.

This research aims to bridge this knowledge gap by synthesizing and analyzing the molecular and electronic properties of 4-((4-((4-aminophenyl)sulfonyl)phenyl)diazenyl) phenol (AZO) using DFT-based computational analysis and SwissADME pharmacokinetic predictions. Additionally, the study explores the incorporation of AZO into a PCL/modified starch polymer nanocomposite, investigating its structural, thermal, and mechanical enhancements through experimental techniques such as X-ray diffraction (XRD) and transmission electron microscopy (TEM)^16–19^.

The combined use of experimental and theoretical approaches has become a key methodology in materials science, offering comprehensive insights into structural transformations across multiple scales. XRD and TEM analyses provide a macroscopic perspective on interlayer spacing, morphology, and structural organization, which is essential for characterizing nanocomposites. However, these techniques alone cannot fully capture the molecular-level interactions driving structural modifications. To overcome this limitation, DFT-based topological analyses, including reduced density gradient (RDG)/non-covalent interaction (NCI) and electron localization function (ELF) calculations, provide visual and quantitative descriptions of weak interlayer interactions that directly impact nanocomposite stability and dispersion.

In this study, a synergistic approach is employed, where XRD and TEM experiments examine the intercalation and exfoliation phenomena of AZO-modified Na-Bentonite, while DFT-based RDG/NCI and ELF analyses elucidate the nature of interlayer interactions at a molecular level. This methodology not only enhances our understand.

## Materials and methods

Dapsone and phenol were supplied by Fluka (Germany, 99%), while sodium bentonite was sourced from Sigma Aldrich (Germany, 99%). Polycaprolactone (PCL) was 99%. Chloroform was sourced from Merck (Germany, 98%). HCl, NaOH, AgNO_3_, and NaNO_2_ were supplied (B.D.H., 98%), and glycerol was obtained from Sigma Aldrich (99%).

### X-ray Diffraction (XRD) analysis

The XRD study was conducted utlizing a Shimadzu XRD 6000 diffractometer with Cu K radiation (k = 0.15406 nm). The diffractogram was scanned in the range from 2θ to 10θ at a scan rate of 1 degree/min.

### Fourier-Transform Infrared (FT-IR) spectroscopy

The FTIR spectra of the blend sample were recorded using a Perkin Elmer FT-IR Spectrum BX (USA) spectrophotometer with the KBr disc technique.

### Transmission Electron Microscopy (TEM)

The dispersion of clay was studied using energy-filtering transmission electron microscopy (EFTEM). TEM images were taken with a LEO912AB Energy Filtering Transmission Electron Microscope at an acceleration voltage of 120 keV. The specimens were prepared using an Ultracut E (Reichert and Jung) cryo-microtome. Thin sections of approximately 100 nm were cut with a diamond knife at 120 °C.

### Synthesis of azo compound

**Fig. 1:**
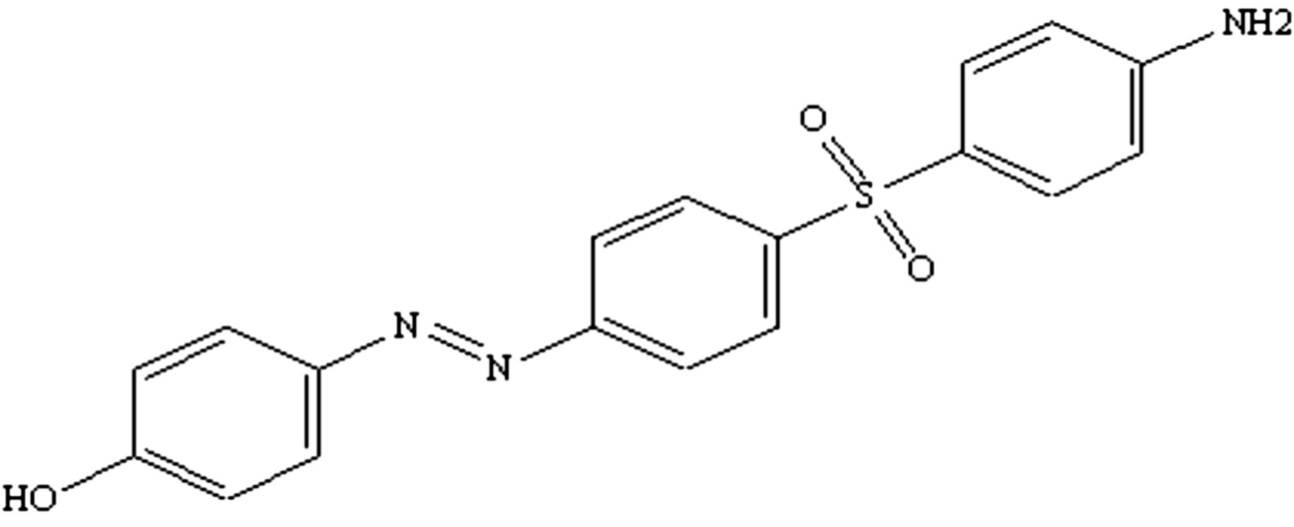
Structure of azo compound, 4-((4-((4 aminophenyl) sulfonyl) phenyl) diazenyl) phenol**.**

The process of synthesizing the azo compound involved several steps. Initially, 4 g of dapsone (equivalent to 0.016 mol) was dissolved in 3 mL of concentrated hydrochloric acid. This mixture was then cooled in an ice bath at temperatures ranging from 0 to 5 ºC before adding 4 to 15 mL of distilled water. Following this, a solution containing sodium nitrite (0.016 mol) (1.1 g) in 5 mL of distilled water was prepared and chilled to the same temperature range. Gradually, distilled water was added to the diazonium salt solution, incorporating a sodium hydroxide solution containing phenol (0.016 mol) (1.5 g) dissolved in 10 mL of pure water^20^, all maintained at temperatures between 0 and 5 °C. After allowing the mixture to stand for a day to facilitate precipitation, it was subjected to cleansing and multiple washes with distilled water to ensure purity. To determine the proportion of the product (53%) in Fig. 1, it underwent further cleansing and washing with distilled water during this period before undergoing recrystallization with ethanol.

### Production of organic clay

The process entails the utilization of bentonite to exchange the ammonium alkyl ion with an azo compound. Initially, 600 mL of filtered water was thoroughly mixed with 4.0 g of sodium bentonite for one hour. Subsequently, a certain volume of azo compound suspension was added to the resulting mixture as an additive. The clay suspension containing the azo compound was then combined with 4.50 g of azo compound in 400 mL of water, followed by the addition of concentrated hydrochloric acid (Emad ^21^). After undergoing vigorous stirring at 80 ºC for one hr., the suspension of organic clay was filtered and washed with water. It was observed that one mole of AgNO_3_ solution reacted with Cl^-^. Following this, the organic layer of the particles was dried for 72 h at 60 ºC.

### DFT methodology

In order to provide fully optimized geometrical and electronic calculations using the hybrid B3LYP technique^22^ , the synthesized heterocyclic compound was subjected to a computational analysis using density functional theory (DFT), and the basis set 6–311 +  + G (d,p) was utilized with Gaussian 09 software^23^. MEP analysis was conducted to determine the significant nucleophilic and electrophilic sites for the optimized structures, and the best structure of the heterocyclic compound, along with its energy excitation levels, was displayed using Chemcraft^24^ and VMD^25^ software to study electronic behavior. The 6–311 +  + G (d,p) harmonic vibrational modes were calibrated for vibrational data using the 0.967 multiplying vibrational scale factor and were found to be acceptable in comparison to experimental results^26^. TD-DFT calculations were performed to estimate the excitation energy levels for different excited states. Computational NMR calculations were carried out using the GIOA method^27^ at the same optimization level. Using Multiwfn software, additional topological studies on reduced density gradient/non-covalent interaction (RDG/NCI) were conducted to illustrate the types of intramolecular interactions and the electron localization function (ELF) to enhance the understanding of interaction types and bond nature between atoms in the investigated heterocyclic system^28^. Using the following formulas, one may determine the estimated quantum reactivity parameters^29^.

The current manuscript will clarify that the RDG/NCI analysis was employed as a qualitative method to identify NCIs, addressing this concern.When paired with RDG and ELF analysis, B3LYP offers a good approximation for visualizing weak interactions, although not being optimal for dispersion-dominated interactions. Higher-level theoretical techniques might be used in future research to increase the precision of NCI predictions.

1 $${\text{E}}_{{{\text{GAP}}}} \, = \,{\text{E}}_{{{\text{LUMO}}}} - {\text{ E}}_{{{\text{HOMO}}}}$$

2 $${\text{I}}\, = \, - {\text{E}}_{{{\text{HOMO}}}}$$

3 $${\text{A}}\, = \, - {\text{E}}_{{{\text{LUMO}}}}$$

4 $$\eta \, = \,\left( {{\text{I}} - {\text{A}}} \right)/{2}$$

5 $$\mu \, = - \left( {{\text{I}}\, + \,{\text{A}}} \right)/{2}$$

6 $$\sigma \, = \,{1}/ \, \eta$$

7 $$\chi \, = \, - \left( {{\text{E}}_{{{\text{HOMO}}}} \, + \,{\text{E}}_{{{\text{LUMO}}}} } \right)/{2}$$

Where E_Gap_, Energy gap(E_GAP_ = E_LUMO_ − E_HOMO_, a large E_Gap_ indicates a stable and less reactive molecule (e.g., insulating materials). A small E_Gap_ suggests a more reactive molecule (e.g., conductive or semiconducting materials). I, Ionization energy (I =  − E_HOMO_​, the higher the HOMO energy (closer to 0 eV), the easier it is to lose an electron. A, Electron affinity(A =  − E_LUMO_​, the lower (more negative) the LUMO energy, the easier it is for the molecule to accept an electron. Η, Chemical hardness(η = $\frac{1-A}{2}$, Large η → more stable, less reactive while small η → more reactive, easily polarizable. Hard molecules have a large HOMO–LUMO gap, while soft molecules have a small HOMO–LUMO gap). μ Chemical Potential( μ=$\frac{1+A}{2}$ , negative μ suggests that the molecule is more stable and less likely to lose electrons. $\sigma$, Chemical Softness ($\sigma$ =$\frac{1}{\upeta }$ Large σ → Soft molecule, highly reactive. Small σ → Hard molecule, less reactive). Χ, Electronegativity (χ=$\frac{\text{E HOMO}+\text{E LUMO}}{2}$ High χ → Strong electron-attracting tendency (electrophilic), low χ → Weak electron-attracting tendency (nucleophilic).

### Molecular docking process

Using ligand affinity probabilities and competition between several docked poses, molecular docking was used to find a target protein with possible anti-scab activity. To guarantee precision, the target protein was chosen using docking parameters that were tuned. K. Fischer used an improved azo molecule as a receptor within the active site of the enzyme to analyze the crystal structure of the macromolecule *3H7O*, resolved at 1.85 Å. This method made it possible to assess the hypothesized relationship between binding strength and anti-scab activity. An established reference inhibitor that targets the active site of the scabies mite inactivated protease analog S-I1 (SMIPP-S-I1) was used as a standard for docking validation. The potency of the inhibitor was evaluated by measuring the target protein’s binding affinity.

iGemdock 2.1 was used for molecular docking simulations. After removing unnecessary ligands, ions, and water molecules from the protein, polar hydrogens were added, and Gasteiger charges were assigned. The ligand’s internal energy had the biggest impact on the docking score function, which determined the binding mode and site. To enhance docking accuracy and optimize binding site prediction, the GemDock scoring method was utilized, which incorporates a multi-identified potential energy model . Recent studies have demonstrated the reliability of these methodologies in predicting binding interactions and validating docking performance^30,31^.

Chimera 1.13.1 and BIOVIA Discovery Studio Visualizer v21.1.0 were utilized to visualize and examine unbound interactions. The Protein Data Bank provided the crystal structure of the inactivated protease analog from a monkey mange mite. To learn more about the produced compound’s binding stability, molecular dynamics simulations were run. The solvent environment was represented by the TIP3P BOX model, which was followed by charge neutralization and energy minimization. A 100 ns simulation was run on each system to assess interaction dynamics and structural stability^32–34^. These simulations have been widely adopted in recent computational studies to explore ligand-receptor binding mechanisms with high accuracy^32–34^.

### Preparation of nanocomposite PCL/Starch/AZO-OBNT

A mixture consisting of starch, glycerol, and water was prepared with a mass ratio of 4:0.5:3. PCL dissolution was performed using 50 mL of starch chloroform. Subsequently, the solution of PCL was continuously mixed while gradually adding the AZO-OBNT solution drop by drop. Once the complete solution, containing both starch and AZO-OBNT, was obtained, it was added to the PCL solution and mixed for an hour to facilitate the incorporation of clay (AZO-OBNT) into the PCL/starch matrix. Following this step, the solutions of AZO-OBNT and PCL/starch were thoroughly mixed, and the resulting nanoparticle was cast into Petri dishes^35^. The amounts of AZO-OBNT, PCL/starch and the modified clay utilzed in this study as shown in Table 1.

**Table 1:** A comprehensive summary of the quantities of AZO-OBNT, PCL/starch and organoclay utilized in the present study.

Trial	PCL Mass (g)	Starch Mass (g)	organoclay Mass (g)
1	3	1	0.00
2	2.96	0.99	0.05
3	2.92	0.98	0.10
4	2.88	0.97	0.15
5	2.84	0.96	0.20
6	2.80	0.95	0.25

### The assessment of ADME for the AZO compound designed

In Swiss ADME was utilized to predict the pharmacokinetic profile, encompassing absorption, distribution, metabolism, and excretion, alongside additional aspects like blood–brain barrier permeability, P-gp affinity, and bioavailability. This method aids in the identification of the most secure and auspicious drug candidates while eliminating compounds with unfavorable properties of ADME that are more prone to failure in subsequent stages of drug development^36^**.** The construction of the chemical structures of the newly synthesized AZO compound was accomplished using the Chem Draw tool. Subsequently, the nomenclature of these structures through the Simple molecular input line entry system (SMILES) initiated the process of prediction. Finally, the polarity and lipophilicity of the compounds were evaluated through the Boiled Egg test^37^**.**

## Results and discussion

### Geometrical structure and Frontier Molecular Orbitals (FMOs)

**Fig. 2:**
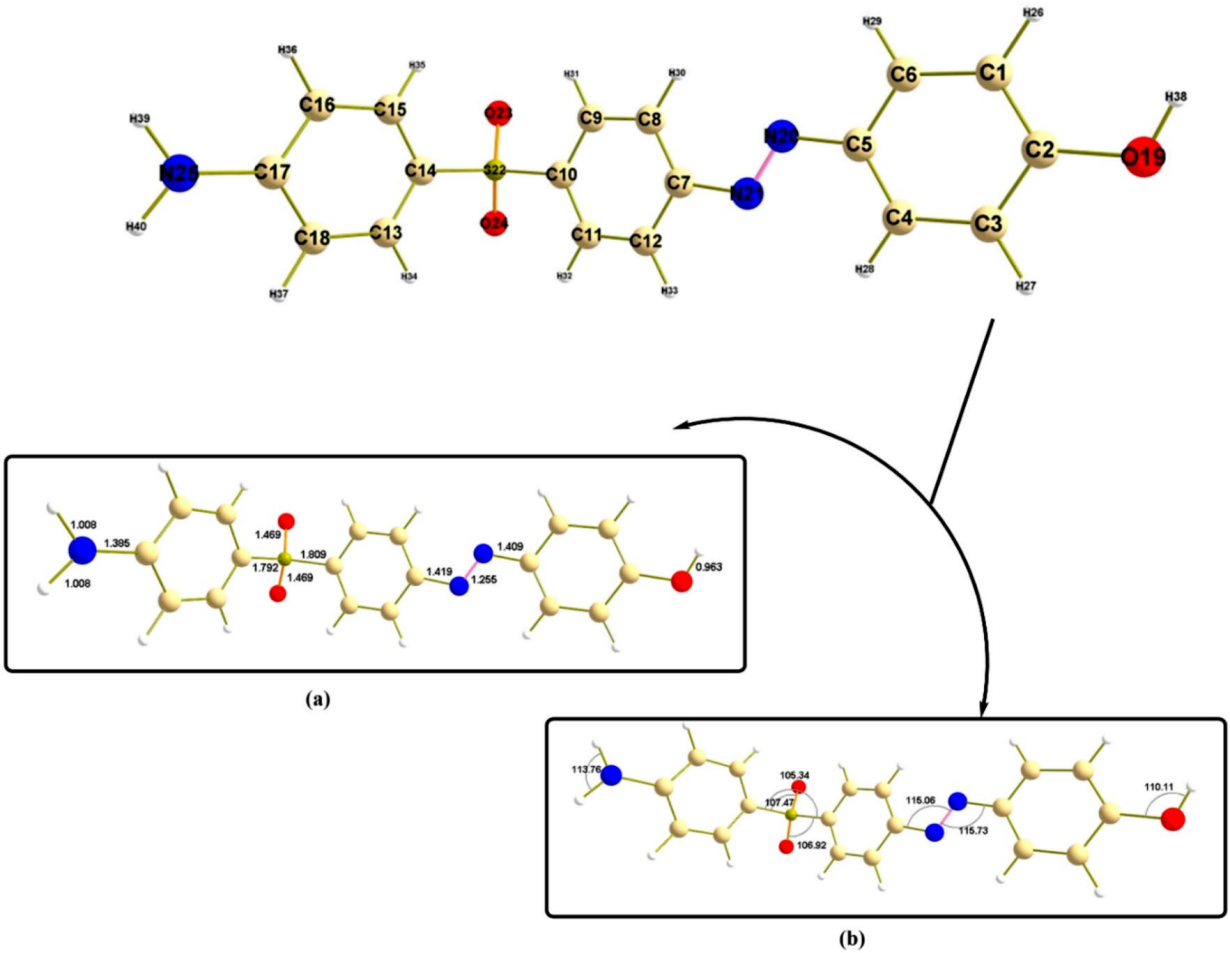
Geometrical structure of the designed compound labeled with, (**a**) bond lengths, (**b**) bond angles.

At the same theoretical level, Fig. 2 presents the geometrical structural convergence of the heterocyclic compound under investigation in its ground state. It is possible to address the fundamental structural parameters bond lengths and bond angles to gain a well understanding of the conformational behavior of the titled structure. The optimized structure appears to be similar in atomic planarity to achieve the best energetic configuration. The bond length of S22-O23 is the same as that of S22-O24 (1.469 Å), while the bond length of C10-S22 (1.809 ºA) slightly differs from the value of C14-S22 (1.792 Å). This can be attributed to the surrounding environment, which mainly involves the link of contact for both C10 and C14. The same finding is observed in C5-N20 (1.409 Å) and C7-N21 (1.419 ºA). Planarity behavior between atoms can be elaborated upon as a result of the bond angle values of the optimized structure; in the case of C5-N20-N21, the bond angle value (115.73°A) is similar to that of C7-N21-N20 (115.06°A), while there is a difference between the bond angles of C10-S22-O24 (106.92°A) and C14-S22-O23 (107.47°A).

**Fig. 3:**
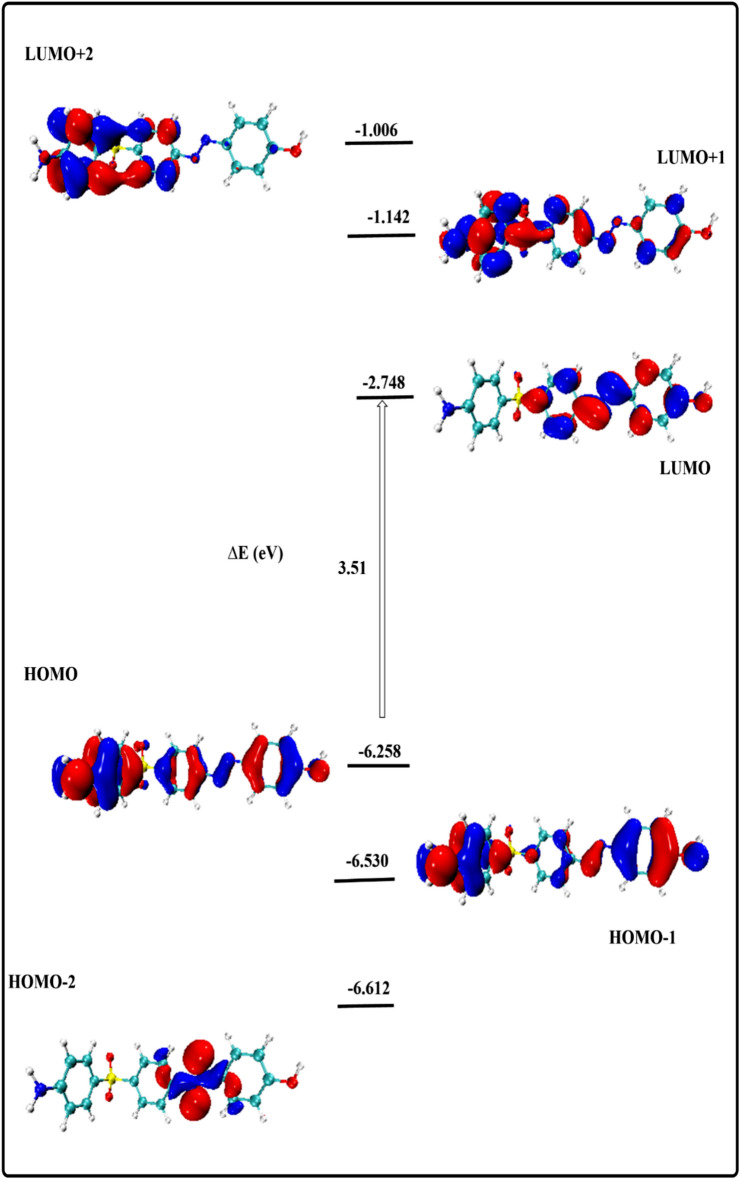
Energy excitation levels with energy values (eV) software >  > computational programs were visualized using VMD 1.9.3 and Multiwfn 3.7. Accession Number: [ 10.1002/aoc.5800] [https://zenodo.org/records/14991491].

In order to predict the stability and reactivity of different molecules, it is essential to study the properties of the compounds’ frontier molecular orbitals (FMOs)^38^. Figure 3 displays the energy distribution of the most significant molecular orbitals (HOMO-2, HOMO-1, HOMO, LUMO, LUMO + 1, and LUMO + 2) for the optimized gaseous structures. The stability of the current heterocyclic complex can be predicted using the amplitude of the energy gap (∆E = 3.51 eV) between the HOMO and LUMO levels, a crucial parameter in the study of stability and reactivity^39^. The orbital contribution at all examined levels was mostly observed throughout the proposed molecule, providing additional evidence for the stability of both the ground and excited states. The majority of scientific fields involving interacting systems can be analyzed using several important quantum reactivity indices, which are also connected to biological processes. Based on the FMO energy values, physicochemical and reactivity parameters are created to provide details about the electronic characteristics and stabilities of the complexes under study. The metrics that best describe molecular properties are ionization potential (I), electronegativity (χ), chemical potential (µ) global softness (S), and chemical hardness (η), electron affinity (A) as shown in Table 2. It is commonly recognized that a chemical system becomes m ore stable as the HOMO–LUMO gap (EGAP, ∆E) increases. A and I are reversible properties because the more nucleophilic system has a higher ionization potential and a lower electron affinity. The nucleophilic-electrophilic ability indices showed a stable designed structure with lower I and lower A when compared to earlier studies. Furthermore, a less polarizable chemical system will lead to less solvent stabilization, as indicated by the smaller σ and greater η.

**Table 2:** Quantum chemical parameters of the studied compounds in gas phase.

Parameters	Compound
E_HOMO_ (eV)	-6.258
E_LUMO_ (eV)	-2.748
ΔE (eV)	3.51
E (a.u.)	-1483.283
D (debye)	7.218
I (eV)	6.258
A (eV)	2.748
µ (eV)	-4.503
η (eV)	1.755
σ (eV)	0.570
χ (eV)	4.503

### IR spectral analysis

**Fig. 4:**
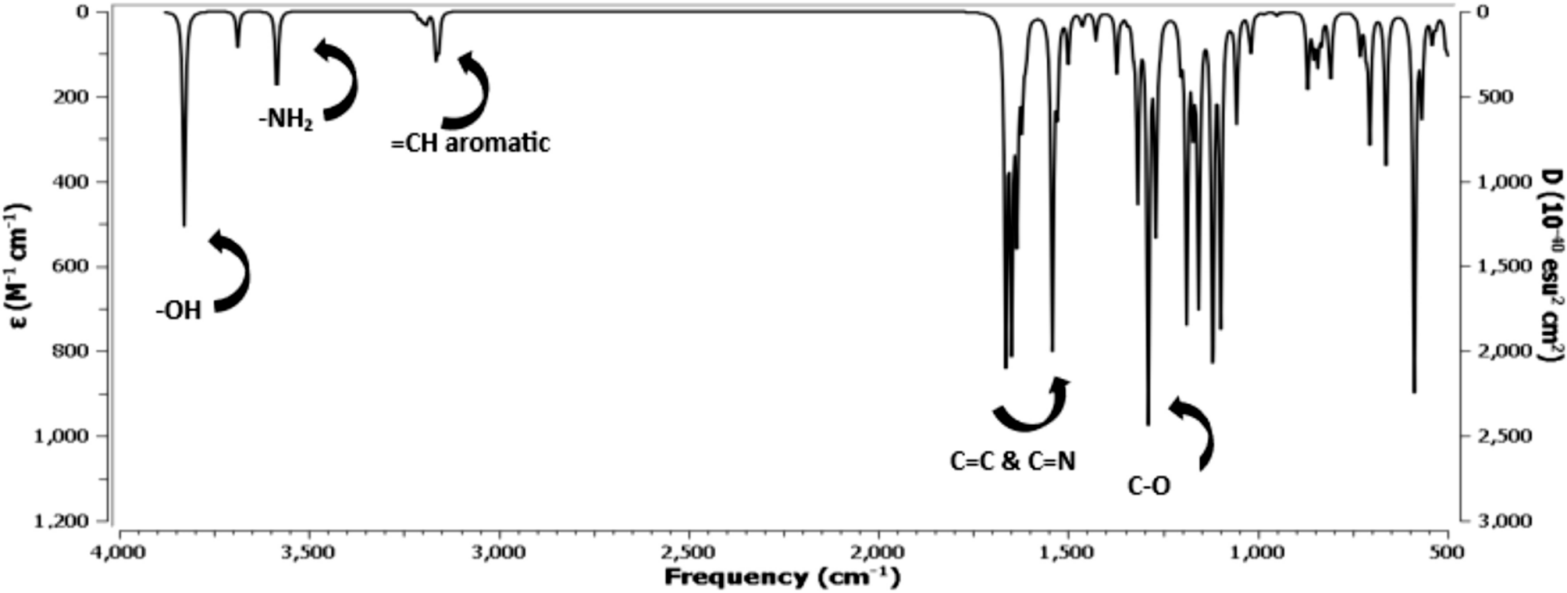
Infrared spectrum structure of the chosen Azo compound with DFT/B3LYP/6-311G (d,p) way.

**Fig. 5:**
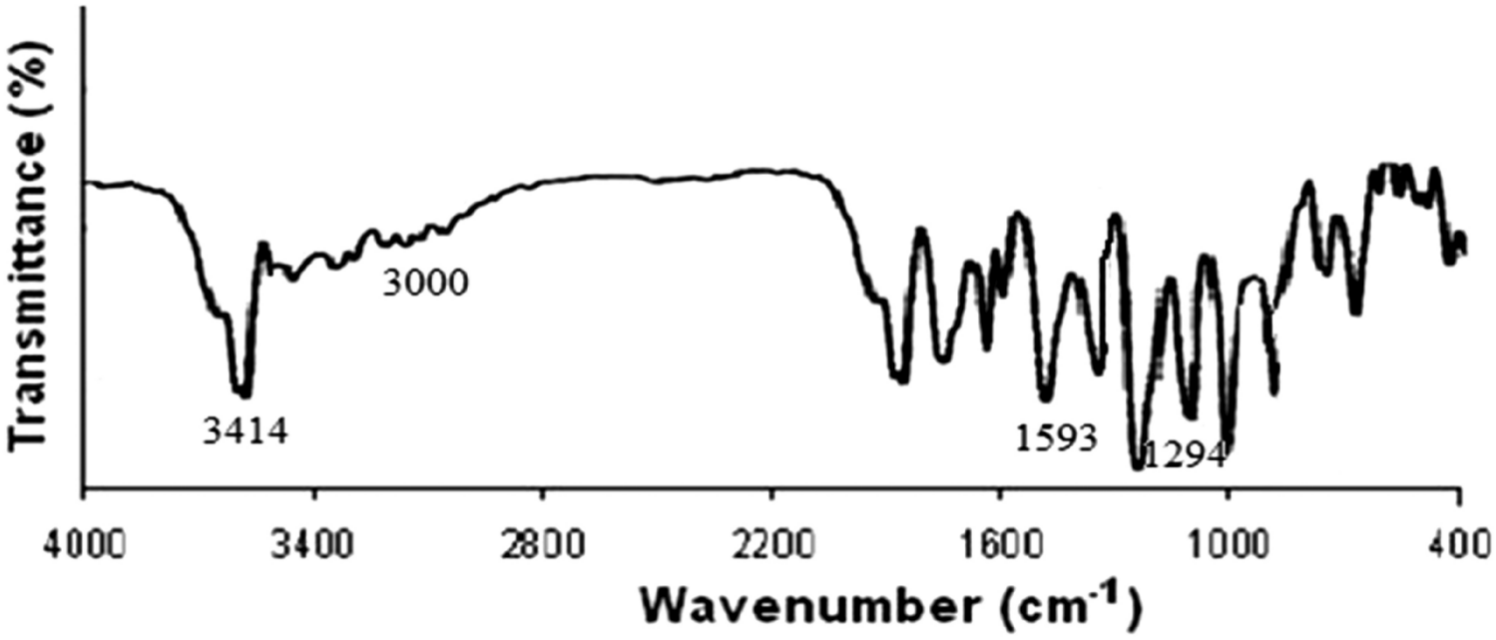
Infrared spectrum of azo compound practically.

The molecular structures of the studied azo compounds were confirmed through the calculation of their vibrational spectra using IR spectral analysis. Quantum mechanical methods are primarily based on harmonic potentials; therefore, a correction step should be performed before comparing experimental results with computational results. It was estimated that the data investigated should be multiplied by a scale factor of 0.967 to calibrate with the experimental results. Table 3 presents the data corrected with the specified scale factor for the B3LYP functional and also includes the corresponding experimental data for each studied ligand. Figure 4 depicts some characteristic bands for the azo compound. A sharp band observed at 3687–3584 cm⁻^1^ corresponds to the stretching of the NH₂ bond in the azo, while the bands for NH₂ appear at 3565 cm⁻^1^ and 3465 cm⁻^1^. The combined practical value of O–H with NH₂ in the azo appears at 3414 cm⁻^1^. The disparity between the solvent environments used in the preparation process may account for the differences between theoretical and experimental data. The computational study was conducted in pure ethanol rather than in the solid state. The stretching of the C-H bond in the aromatic moiety is responsible for another band that appears at 3212 cm⁻^1^ and 3157 cm⁻^1^, which theoretically should appear at 3000 cm⁻^1^. The C = C group is represented by a strong, crisp band at 1649–1621 cm⁻^1^, corresponding closely to the experimental value of 1593 cm⁻^1^. The N = N group is represented by experimental bands at 1398 cm⁻^1^, while the predicted N = N azo band occurs at 1428 cm⁻^1^. The presence of the C = N groups is indicated by the band observed at 1649 cm⁻^1^. A band at 1593 cm⁻^1^, which corresponds to the C = N group, aligns somewhat closer to the experimental finding than other results. Although theoretically it should appear at 1288 cm⁻^1^, the experimental IR C-O band emerges at 1294 cm⁻^1^.Figure 5

**Table 3:** Calculated frequencies from B3LYP/ 6–311 +  + G(d,p) and experimental IR frequencies.

Functional group	Frequency B3LYP/LANL2DZ/6-311G(d,p) (cm^-1^)	Frequency x scale factor (cm^-1^)	Experimental Frequency (cm^-1^)
OH	3828	3701	3414
NH_2_	3687–3584	3565–3465	3414
= C-H (aromatic)	3212–3157	3106–3052	3000
C = C	1649–1621	1594–1567	1593
C = N	1649	1594	1593
C-O	1288	1245	1294

### UV–vis electronic spectra by TD-DFT method

**Fig. 6:**
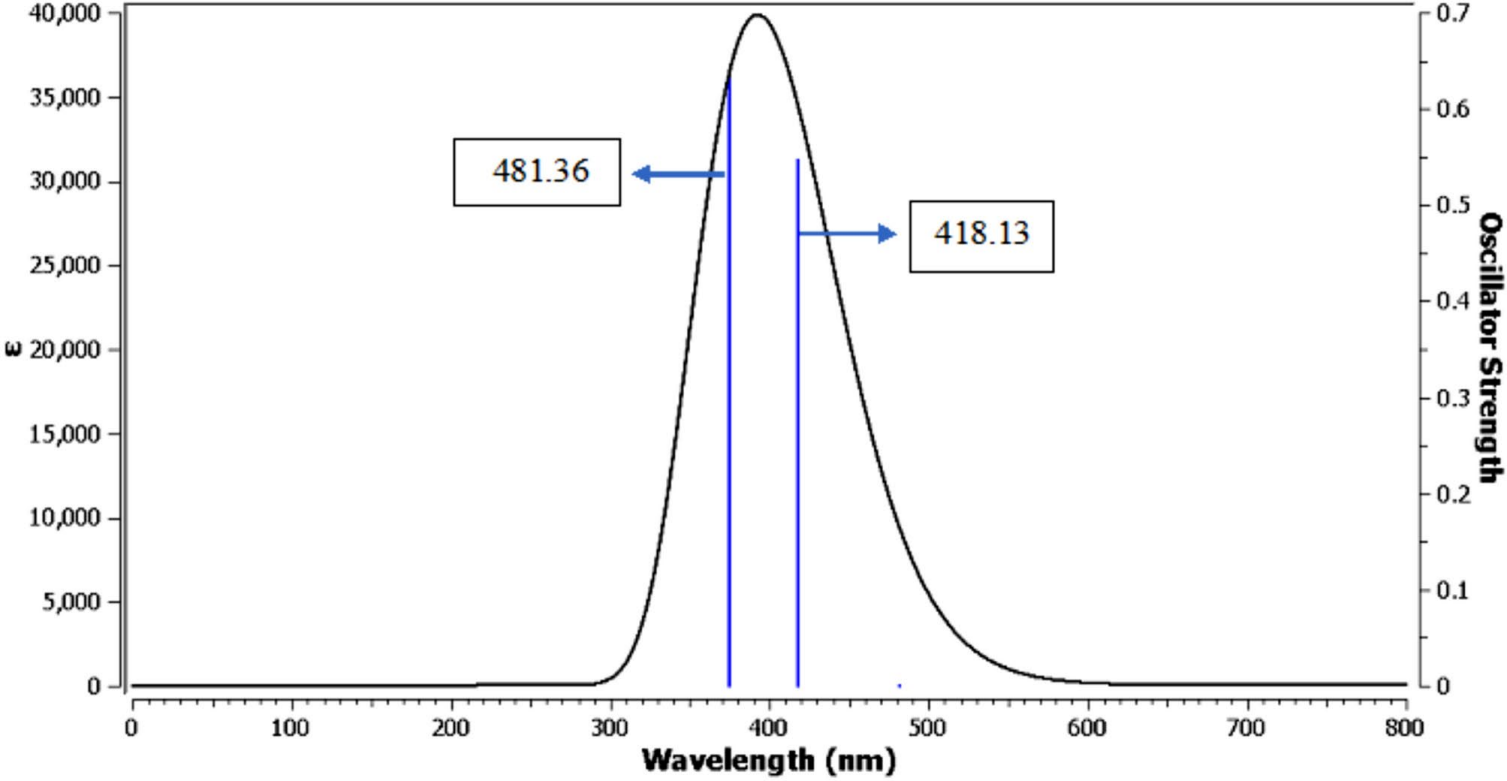
UV–Vis electronic absorption spectra for the designed compound.

The electrical behavior of the studied compound was described using TDDFT and CPCM as the solvation model. The TD-DFT computations were performed using the default Gaussian 09 parameters. To analyze six states, the software settings were changed to Nstate = 6. Three transition bands are seen as lines in Fig. 6. The first transition, which corresponds to the π-π* transition, is represented by a weak singlet absorption band in the Gaussian calculation’s LOG file, with λmax = 481.36 nm and a very weak oscillator strength (F) of 0.0001. This orbital contribution includes HOMO-2 to LUMO. The other two transitions appear stronger for the HOMO to LUMO and HOMO-1 to LUMO contributions, with λmax values of 418.13 nm and 374.59 nm, respectively, as shown in Table 4. These strong spectral lines correspond to π-π* and n-π* types.

**Table 4:** Excitation energies, maximum wavelengths, oscillator strengths and % orbital contribution for the designed compound**.**

The number of spectral line	Excitation energy (eV)	λ_max_ (nm)	F	Transition type	% orbital contribution
1	2.756	481.36	0.0001	HOMO-2 → LUMO	70.1
2	2.965	418.13	0.547	HOMO → LUMO	70.6
3	3.310	374.59	0.635	HOMO-1 → LUMO	70.4

### NMR spectral analysis

**Fig. 7:**
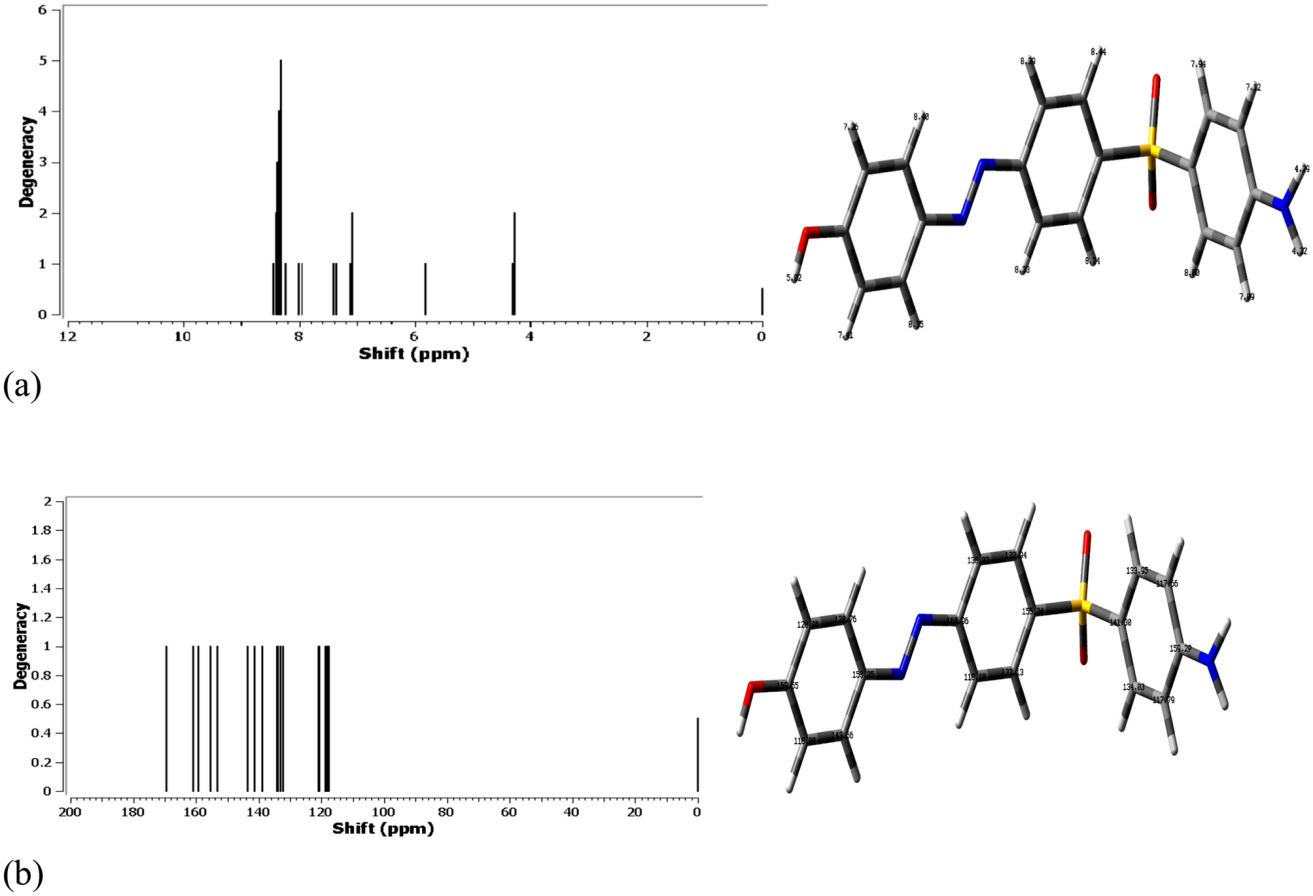
**S**pectr a of the designed structure via GIAO method on the optimized heterocyclic structure, (**a**) ^1^H NMR (**b**)^13^C NMR.

The GIAO method was applied to the optimized compounds using the 6-311++G (d,p) basis set in order to compare the theoretical and experimental NMR spectral analyses. Figure 7 shows the ^1^H and ^13^C NMR spectra of the created heterocyclic molecule. The ^1^H NMR Ar-H peak of the heterocyclic compound ranges from 7.09 ppm to 8.44 ppm, depending on the chemical shift (δ) and shielding effect. This range is compared to the experimental value of 9.41 ppm. The proton of the hydroxyl group was found to exhibit a spectral line around 5.82 ppm. The ^13^C NMR computational results, which vary for aromatic rings from 117.66 ppm to 159.29 ppm, are also shown in Fig. 7b.

### Electron Localization Function (ELF)

**Fig. 8:**
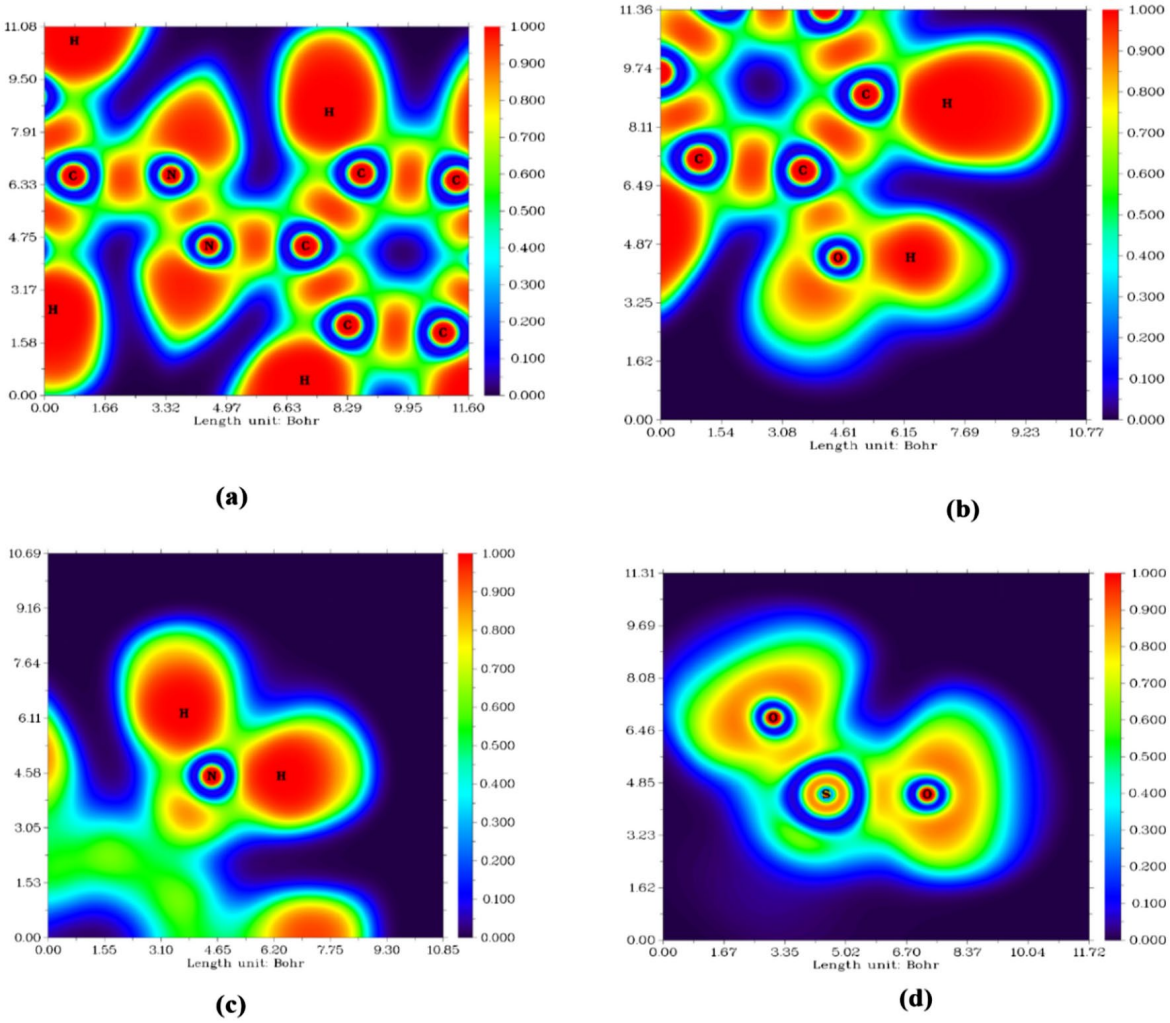
Electron localization function (ELF) colored map of heterocyclic compound (**a**) C5-N20-N21, (**b**) H38-O19-C2, and (**c**) H40-N25-H39, and (**d**) O24-S22-O23.

ELF explores the empirical concepts of electron localization, especially the localization of electron pairs, in the spirit of Lewis structures. An electron localization factor (ELF) in atomic space designates a point where electron confinement and bond type are known. The most important 2D planes in the compound were visualized to predict the nature of the bond and the degree of planarity between atoms, aiming at electronically localized areas. The C5-N20-N21, H38-O19-C2, H40-N25-H39, and O24-S22-O23 planes were demonstrated, as displayed in Fig. 8. The selected planes estimate a localized electronic area (red color scale) between C and N (Fig. 8a), as the electronic cloud deformation appears on the N atom due to the lone pair of electrons^40^. While the N = N bond appears as a localized electronic area demonstrating equal sharing of electrons, the bond between C and O describes a major ionic behavior, as the electronic area appears delocalized around O. Furthermore, the amino group appears out of the molecular plane, as displayed in Fig. 8c. The SO_2_ group was also exported out of the molecular plane due to its atoms.

### Molecular Electrostatic Potential (MEP)

**Fig. 9:**
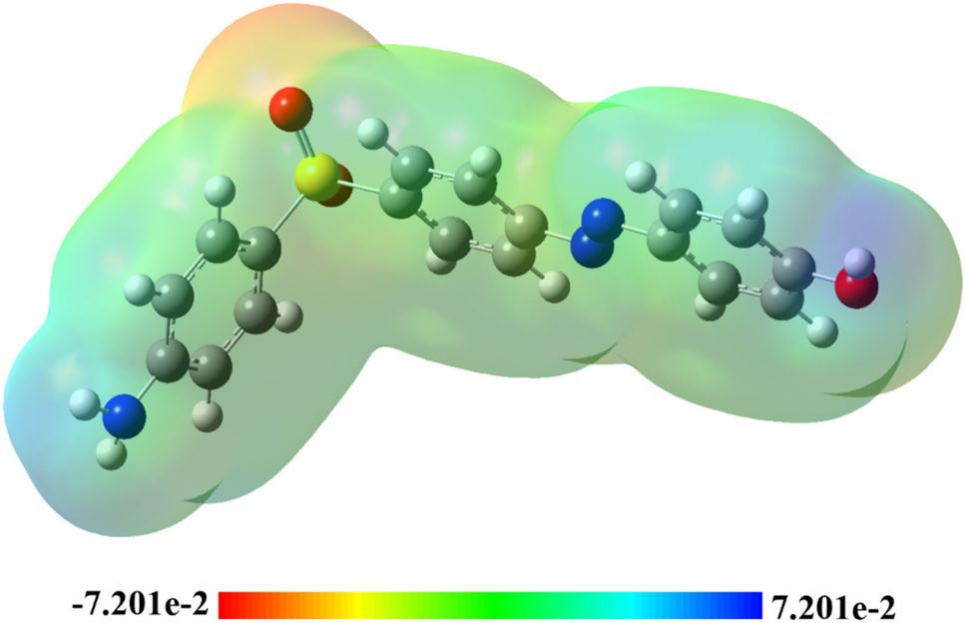
3D-colored map of molecular electrostatic potential of the designed compound software >  > computational programs analysis was conducted and visualized using gauss View09. Accession Number: [ 10.1002/aoc.5800] [https://zenodo.org/records/14991491].

The molecular electrostatic potential (MEP) 3D map is used to analyze the electronegativities of atomic locations in molecules. This topological index helps explain molecular contacts and recognition processes, as long-range interactions are primarily caused by electrostatic forces^40^. The structures of the studied compounds were visually evaluated using colors such as red, orange, yellow, green, and blue to identify potential sites for electrophilic or nucleophilic attacks (Fig. 9). The following order of colors represents the decreasing potential for each atomic site: orange, red, green, yellow, and blue. Consequently, an electron-rich site is indicated by a red zone, while an electron-deficient site is indicated by a blue zone. More specifically, it was predicted that the oxygen atoms of the SO_2_ group in the created compound would have the maximum electronic richness, with the remaining portion of the molecule striving to reach a neutral state and containing only a small amount of π-electrons from the phenyl ring (yellow color scale). The electrophilic sites can be observed in the hydrogen atoms of the amino and hydroxyl groups.

### Reduced Density Gradient/Non-Covalent-Interactions (RDG/NCI)

**Fig. 10:**
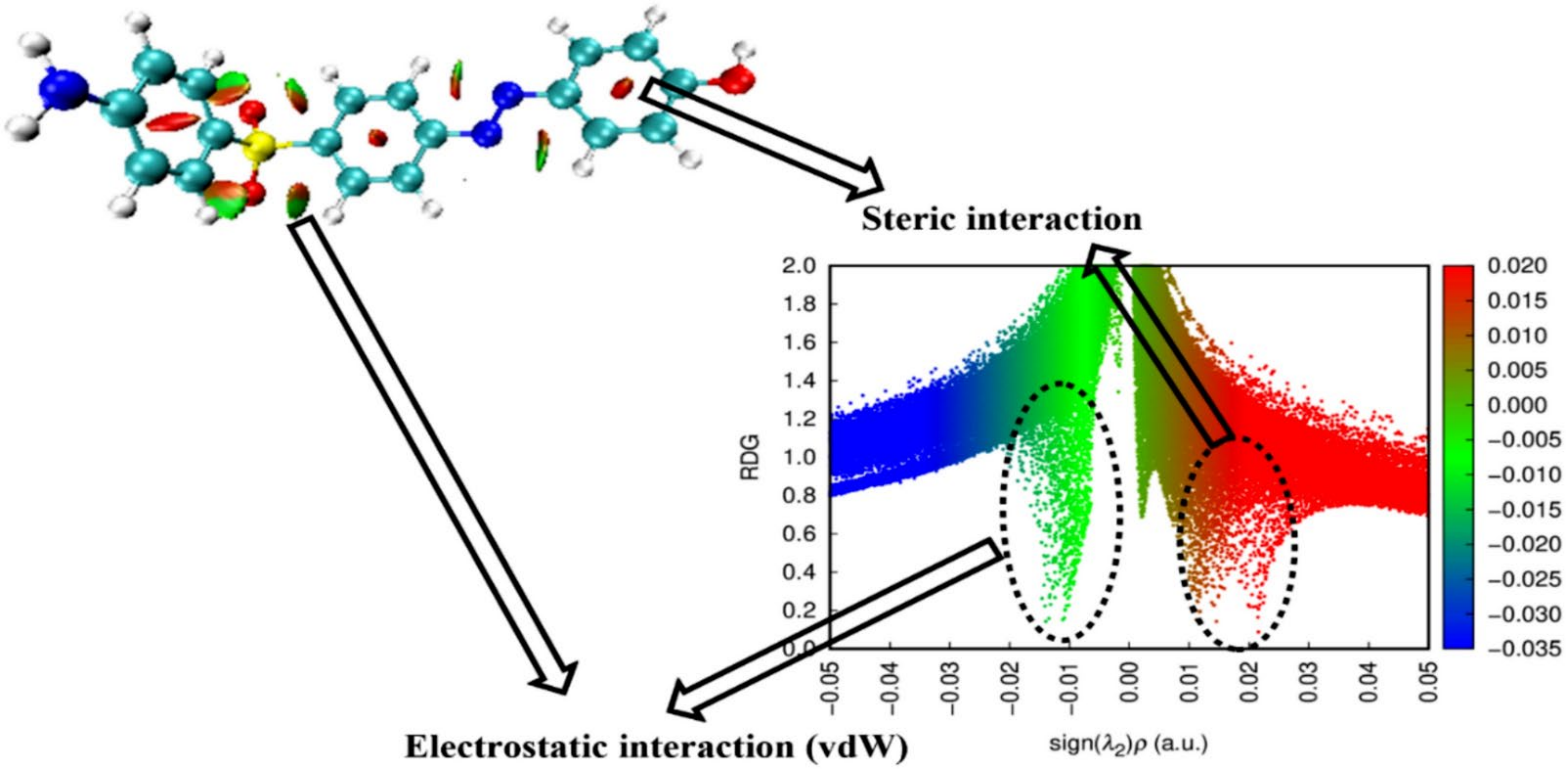
3D-NCI Map and RDG Plot of the designed compound software >  > computational programs were visualized using Multiwfn 3.7 and VMD 1.9.3. Accession Number: [ 10.1002/aoc.5800] [https://zenodo.org/records/14991491].

Utilizing reduced density gradient (RDG) research, noncovalent bond interactions (NCIs) between several molecular sites were identified. Different color codes were used to illustrate the various noncovalent interactions. As illustrated in Fig. 10, the types of interactions that are readily discernible on the surface of each molecule are van der Waals (vdW) interactions, represented by a green color scale, and repulsion (steric) interactions, represented by a red color scale. The sign (λ2)ρ, obtained by multiplying the electron density by the sign of the second Hessian eigenvalue, indicates the strength of the hydrogen bond (HB) interaction in compounds. In this study, vdW interactions are clearly visible in the cages of SO_2_ and N = N planes; however, the intramolecular structure did not exhibit HB formation. Furthermore, in the presence of an unfavorable repulsive interaction, primarily within the range 0.02 > sign (λ2)ρ > 0.01, a significant vdW spike (van der Waals interactions within the range 0 > sign(λ2)ρ > -0.015) was observed.

### Interpretations of the molecular docking study

**Fig. 11:**
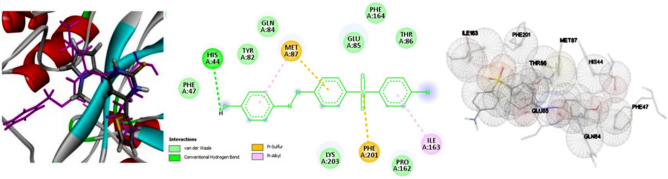
Diagrammatic representations of 3D and 2D ligand-Mpro interactions in the presence of *3H7O*, including conventional hydrogen bond distance values, pi-sulfur interactions, and pi-alkyl interactions of the ligand with an amino acid of *3H7O* from the studied AZO compound [software >  > computational programs discovery studio, Auto Dock 4.2 ]. Accession Number: [ 10.1002/aoc.5800] [https://zenodo.org/records/14991491].

One application of computer-aided design (CAD) is molecular docking, which involves predicting the interactions between two molecules. The small molecule docking method has numerous applications in drug discovery. An example of this method is docking proteins with their respective ligands. In recent times, docking has also been used to forecast interactions between two macromolecules. According to the findings depicted in Fig. 11, the ligand was observed to form two hydrogen bonds with Gln84 and His44, and two ionic bonds with Met87 and Phe201. Additionally, seven van der Waals interactions were identified with Glu85, Phe164, Pro162, Thr86, Tyr82, Phe47, and Ile163.

These interactions are in agreement with recent computational docking studies, which have demonstrated the crucial role of hydrogen bonding and van der Waals interactions in stabilizing ligand–protein complexes^30–34^.Additionally, molecular dynamics simulations confirmed the structural stability of the complex, further validating the observed binding interactions. These findings align with recent literature on docking and molecular simulations, reinforcing the robustness of the adopted computational approach.

### Interpretations of absorption, distribution, metabolism and excretion study

**Fig. 12:**
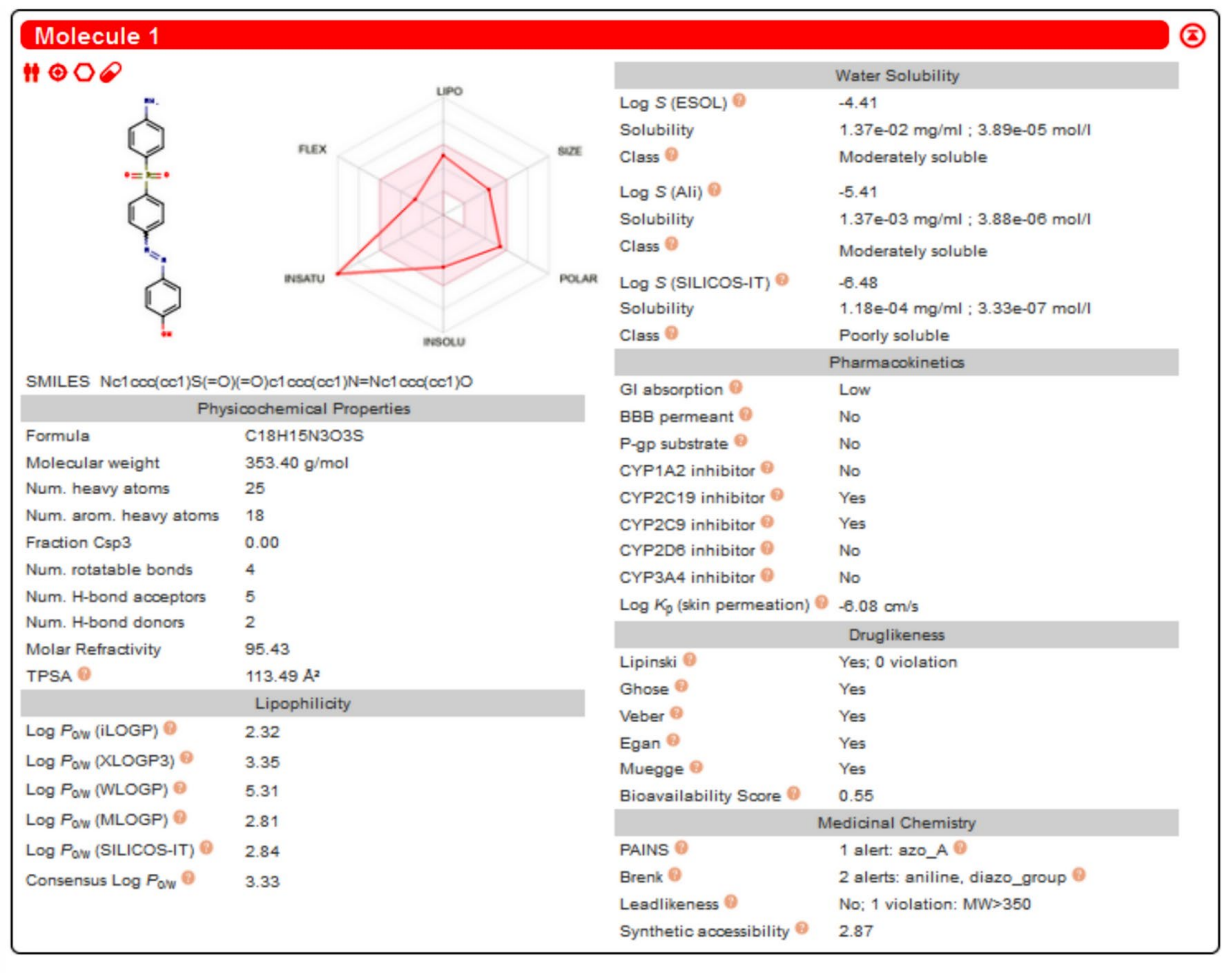
ADME investigation of AZO compound.

**Fig. 13:**
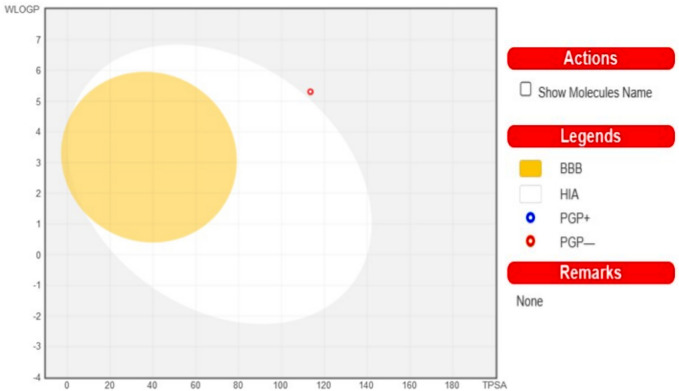
Brain Or Intestinal Estimate D permeation predictive mode-Egg model for AZO compound.

The methodologies utilized in our research involved the Swiss ADME web tool for evaluating pharmacokinetics, drug-likeness, and medicinal chemistry friendliness of compounds. This was done to explore streamlined data management and processing. The Swiss ADME web tool offers free access to a variety of predictive models for physicochemical properties, pharmacokinetics, drug-likeness, and medicinal chemistry friendliness, which include the BOILED-Egg, iLOGP, and Bioavailability Radar methods. Both experts and novices in cheminformatics or computational chemistry can swiftly predict crucial properties of compound groups to assist in drug development endeavors^36^. The concept of drug-likeness emerged to provide valuable insights in the early stages of drug discovery, enhancing the chances of a compound progressing through clinical trials. Drug-likeness represents the cumulative physicochemical properties of medicinal compounds. While commonly linked to pharmacokinetics and safety, it also pertains to molecules with favorable ADMET characteristics. Lipinski’s Rule of Five (Ro5), a renowned guideline introduced in 1997, identifies small molecules suitable for oral absorption based on criteria such as a molecular weight below 500 g/mol, a limited number of hydrogen bond donors, and a specific octanol–water partition coefficient. The pharmacokinetic profile of the AZO compound, including absorption, distribution, metabolism, and excretion, was assessed using the SwissADME tool, alongside parameters like bioavailability, BBB penetration, affinity and Pg. This approach aids in identifying safe and reliable candidate drugs by excluding compounds with unfavorable ADME properties that are prone to failure in later drug discovery phases. Our investigation involved determining nHBA, nRB, HBD, BS, TPSA, Pgp, BBB, iLOGP, GI absorption, and the human oral absorption percentage through this platform. ADME evaluations of the newly developed AZO compound are depicted in Fig. 12, while Table 5 presents drug-likeness and ADME characteristics. The calculated topological polar surface area (TPSA), associated with compound bioavailability, plays a crucial role by indicating gastrointestinal absorption and blood–brain barrier permeation. Compounds with TPSA values above specific thresholds are considered to have poor oral bioavailability. The TPSA values for our created AZO compounds are below the critical threshold. These AZO-derived compounds exhibited a bioavailability score of 0.55, signifying entry into systemic circulation, but they were unable to cross the BBB due to TPSA exceeding 60. The AZO compound demonstrated high gastrointestinal absorption, indicating significant intestinal absorption post-oral administration, unlike other compounds with inferior gastrointestinal absorption. AZO compounds exhibited a diminished attraction towards P-gp, a protein responsible for obstructing the uptake and utilization of medications by cells^37^. Additionally, the formulated AZO compound adhered to the Lipinski rule of five. The SwissADME online platform was also employed to construct the Brain or Intestinal Estimated Permeation predictive model (BOILED-Egg), commonly referred to as the Egan egg graph of AZO innovative chemical compositions. This particular model was devised to forecast the absorption efficiency of a medication in either the brain or the intestines. It facilitates an intuitive assessment of passive gastrointestinal absorption (HIA) and brain permeability (BBB) based on the positioning of the molecule in the WLOGP-versus-TPSA reference framework. Only AZO compounds are capable of being absorbed effectively from the GIT (the light lead area), while none have the ability to penetrate the brain (the yellow region yolk). Moreover, it was predicted that none of these compounds would be subject to active efflux by P-gp (PGP-). The BOILED-Egg model, as illustrated in Fig. 13, was specifically designed for AZO compounds.

**Table 5:** The output parameters of drug-likeness and ADME.

Compound name	AZO
Num. rotatable bonds	4
Num. H-bond accepters	5
Num. H-bond donors	2
Topological polar surface area**(A**^**2**^**)**	113.49
Bioavailability	0.55
Growth inhibition	Low
Blood brain barrier	No
P-glycoprotein	NO
Octanol/water partition coefficient	Yes
Rule of five	2.32

### XRD analysis

**Fig. 14:**
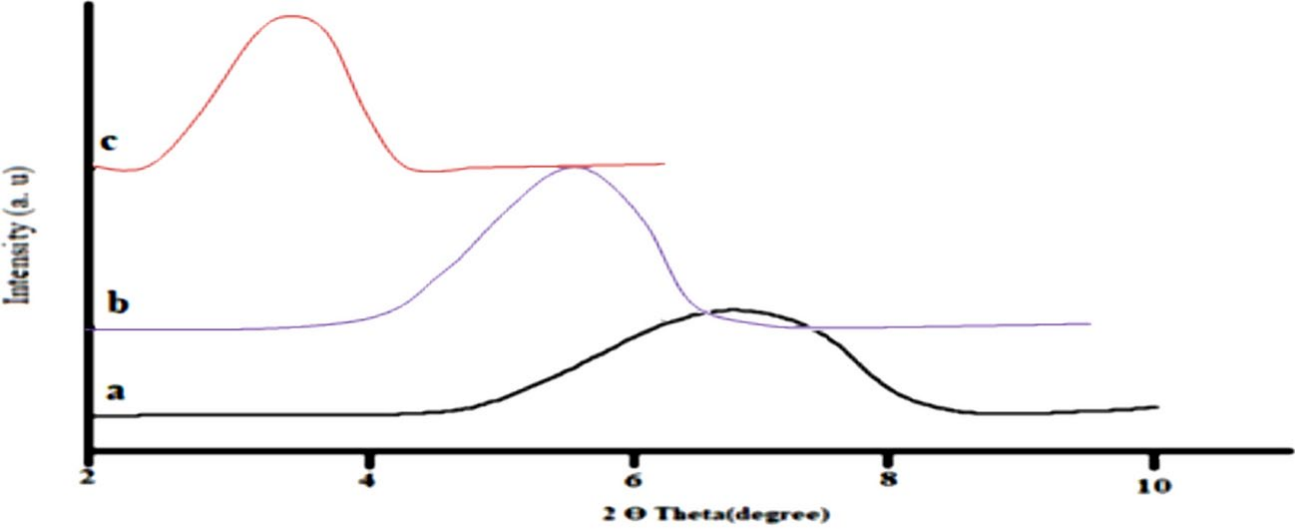
Layer silicate (**a**) Modified Na-BNT, (**b**) Na-BNT by AZO, and(**c**) PCL- AZO-BNT nanocomposite.

**Fig. 15:**
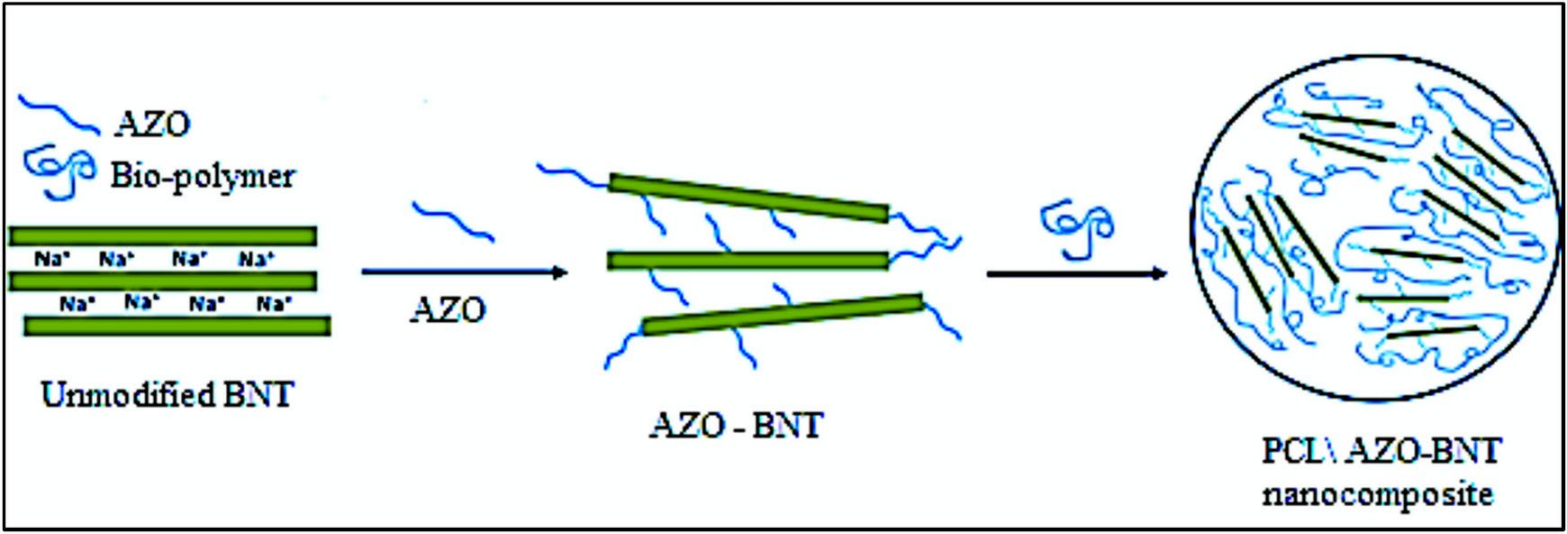
The synthesis of nanocomposites between clay and starch.

The occurrence of AZO-BNT chains in the galleries stimulates the conversion of the original hydrophilic silicate into an organophilic silicate, thereby enhancing the density of the Na-BNT between layers. The increase in density can be explained by Bragg’s law (n = d sin), where “d” represents the distance between two consecutive clay layers and “λ” stands for the wavelength of the X-rays that are diffracted at the incidence angle. According to the X-ray diffraction analysis, Na-BNT exhibits a diffraction peak of d001 at Ө = 7.0762°, indicating that the natural bentonite interlayer distance has a basal spacing of 1.25 nm. To treat the surface of Na-BNT, AZO was employed as a cation-exchanging intercalating agent. Ideally, the cationic head groups of the intercalation agent molecule should be situated at the outer surface layer, while the aliphatic tail extends away from the surface. The elevation in the median basal spacing of AZO-BNT from 1.27 to 1.82 nm(Table 6, Fig. 14) verifies the successful intercalation of AZO into the Na-BNT galleries. A monolayer AZO structure forms within the Na-BNT interlayer gap. The XRD patterns presented in Fig. 15 illustrate the nanocomposites produced with AZO-modified montmorillonite (alkylammonium groups). Table 5 demonstrates that the clay basal spacing experiences an increase of 2.50 nm for 70PCL 30 starch/AZO-BNT. It is observed that the basal spacing of organoclay in the polymer matrix increases proportionally with the size of the surfactant. These XRD patterns indicate that some of the generated nanocomposites are exfoliated, while the majority consist of intercalated compounds as demonstrated in Fig. 1 which provides a representation of the modification of clay with ammonium ions in their nanocomposites. Figure 14 depicts the process of fabricating nanocomposite clay utilizing AZO and PCL/starch clay.

**Table 6:** Diffraction angle and basal spacing of bentonite natural clay (Na-BNT), modified clays with the AZO- BNT and PCL \ Starch -OBNT nanoparticle.

Sample	Exchanged cation	(2Ө°)	d (001) spacing (nm)
Bentonite	Na^+^	7.08	1.25
AZO	C_18_ H_12_ N_2_ O_3_ S NH_3_^+^	4.86	1.82
70PCL \30Starch-AZO-BNT	-	3.53	2.50

### TEM

**Fig. 16:**
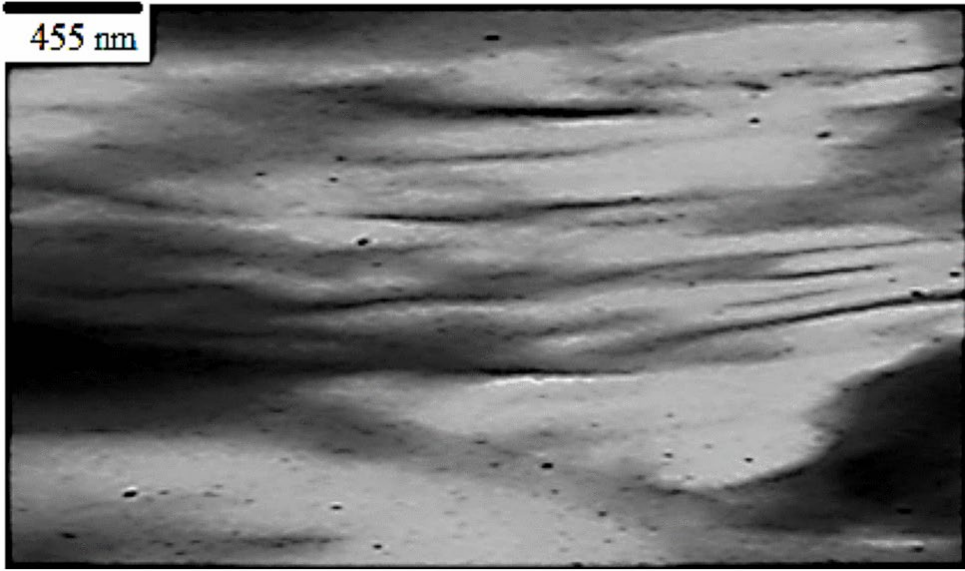
TEM micrograph of biopolymer film filled with 3wt% PCL/Starch-OBNT nanocomposite.

The preservation of the initial Na-MMT stack morphology was achieved by employing PCL/cornstarch, as both components were incompatible, as evidenced by the TEM micrographs of PCL/cornstarch composites shown in Fig. 16^41^. The darkness of the lines (chimneys, licks) corresponds to the thickness of the individual clay layers or clumps. The original layered structure of the organic clay is not observable. Various terms, such as intercalated lamellae, stages with different numbers of lamellae, and pulp aggregates, are used to describe the resulting structure. The TEM micrographs of PCL/cornstarch/AZO-BNT reveal a higher level of intercalation and some areas of exfoliation^42^.

### XRD and TEM analyses and their relation to molecular interactions

X-ray diffraction (XRD) and transmission electron microscopy (TEM) examinations and their correlation to molecular interactions. The XRD analysis indicated an increase in the interlayer spacing of sodium bismuth titanate (Na-BNT) from 1.27 nm to 1.82 nm subsequent to the intercalation of azobenzene (AZO), as demonstrated by a notable shift in the d001 diffraction peak. The observed expansion of the interlayer spacing implies the successful incorporation of AZO molecules within the clay interlayers, thereby resulting in structural enlargement. Likewise, the TEM micrographs corroborated this intercalation phenomenon, exhibiting areas characterized by unique stacking arrangements and partial exfoliation, which underscores the role of interlayer forces in facilitating structural alterations. Despite the clear evidence presented by XRD and TEM regarding structural modifications, these methods do not elucidate the nature of the interlayer interactions that confer stability to these configurations.

In order to further clarify the nature of the interactions that regulate the structural alterations observed, density functional theory (DFT)-based reduced density gradient (RDG)/non-covalent interaction (NCI) and electron localization function (ELF) analyses were conducted. These analytical approaches facilitated the visualization of the non-covalent interactions that are responsible for the stabilization and dispersion of interlayers.

The RDG isosurface representations were used to provide a qualitative assessment of the weak non-covalent interactions present within the azobenzene (AZO)-modified sodium bismuth titanate (Na-BNT) system. The findings revealed the presence of green regions, indicating van der Waals interactions, which dominate within the intercalated system. These interactions help stabilize the AZO molecules embedded within the Na-BNT layers and contribute to the observed interlayer expansion. Additionally, blue regions were concentrated around the functional groups of AZO that interact with the clay substrate, further supporting the intercalation phenomenon. Furthermore, red regions, representing steric repulsion, were observed, which explain the incomplete exfoliation in certain areas.

The electron localization function (ELF) was examined to elucidate the charge distribution and electron delocalization within the interlayer region. The results confirmed the existence of electron density accumulation near the functional groups, indicating charge transfer effects that enhance the interaction between AZO and the Na-BNT layers. A moderate extent of charge delocalization was also observed, which correlates with the observed increase in basal spacing, as evidenced by X-ray diffraction (XRD) results.

These computational insights substantiate the experimental observations from XRD and transmission electron microscopy (TEM), illustrating that van der Waals interactions and localized charge effects are integral to the processes of interlayer expansion and stabilization of dispersion.

## Conclusion

The computational chemistry-based investigation has clarified the electronic and structural characteristics of the synthesized azo compound using density functional theory (DFT). RDG and ELF analyses revealed key non-covalent interactions within the structure, such as van der Waals forces and hydrogen bonding, which contribute to the stability and reactivity of the compound. Spectroscopic analysis further confirmed its stability, while virtual docking tests identified potential interactions with the 3H7O protein. TEM analysis demonstrated partial exfoliation of AZO-BNT nanocomposites upon intercalation, and the study explored new polymeric nanocomposites involving AZO, PCL-modified cornstarch, and BNT, highlighting their promising thermal properties and potential applications in recyclable packaging.

The integration of XRD, TEM, and DFT-based analyses has provided a comprehensive understanding of the AZO-modified Na-BNT system. The study reveals the importance of non-covalent interactions, such as van der Waals forces and hydrogen bonding, in stabilizing the interlayer structure. These findings underscore the value of combining empirical and theoretical approaches in nanocomposite characterization.

For future research, investigating the biological activity of the azo compound, assessing the environmental stability of AZO-based nanocomposites, and optimizing synthesis parameters to improve AZO dispersion in polymer matrices are recommended. These steps will enhance the application potential of AZO-based materials, particularly in sustainable and biodegradable packaging solutions.

## Data Availability

The datasets used and/or analysed during the current study available from the corresponding author on reasonable request.
